# Endurance Training Depletes Antioxidant System but Does Not Affect Endothelial Functions in Women with Abdominal Obesity: A Randomized Trial with a Comparison to Endurance-Strength Training

**DOI:** 10.3390/jcm10081639

**Published:** 2021-04-12

**Authors:** Małgorzata Jamka, Paweł Bogdański, Patrycja Krzyżanowska-Jankowska, Anna Miśkiewicz-Chotnicka, Joanna Karolkiewicz, Monika Duś-Żuchowska, Radosław Mądry, Aleksandra Lisowska, Anna Gotz-Więckowska, Saule Iskakova, Jarosław Walkowiak, Edyta Mądry

**Affiliations:** 1 Department of Pediatric Gastroenterology and Metabolic Diseases, Poznan University of Medical Sciences, Szpitalna Str. 27/33, 60-572 Poznań, Poland; mjamka@ump.edu.pl (M.J.); pkrzyzanowska@ump.edu.pl (P.K.-J.); chotnicka@ump.edu.pl (A.M.-C.); mduszuchowska@ump.edu.pl (M.D.-Ż.); 2 Department of Treatment of Obesity, Metabolic Disorders and Clinical Dietetics, Poznan University of Medical Sciences, Szamarzewskiego Str. 82, 60-569 Poznań, Poland; pbogdanski@ump.edu.pl; 3 Department of Food and Nutrition, Poznan University of Physical Education, Królowej Jadwigi Str. 27/39, 61-871 Poznań, Poland; karolkiewicz@awf.poznan.pl; 4 Department of Oncology, Poznan University of Medical Sciences, Szamarzewskiego Str. 84, 60-569 Poznań, Poland; rmadry@ump.edu.pl; 5 Department of Clinical Auxology and Pediatric Nursing, Poznan University of Medical Sciences, Szpitalna Str. 27/33, 60-572 Poznań, Poland; alisowska@ump.edu.pl; 6 Department of Ophthalmology, Poznan University of Medical Sciences, Szamarzewskiego Str. 84, 60-569 Poznań, Poland; agotzwieckowska@ump.edu.pl; 7 Department of Pharmacology, Asfendiyarov Kazakh National Medical University, Tole Bi Str. 94, Almaty 050000, Kazakhstan; saule1@inbox.ru; 8 Department of Physiology, Poznan University of Medical Sciences, Święcickiego Str. 6, 60-781 Poznań, Poland; emadry@ump.edu.pl

**Keywords:** antioxidant status, oxidative stress, inflammatory markers, cardiovascular risk

## Abstract

Limited data suggested that inclusion of a strength component into endurance exercises might intensify the beneficial effect of training. However, the available data is limited. Therefore, we aimed to compare the effect of endurance and endurance-strength training on anthropometric parameters, endothelial function, arterial stiffness, antioxidant status, and inflammatory markers in abdominally obese women without serious comorbidities. A total of 101 women were recruited and randomly divided into endurance (*n* = 52) and endurance-strength (*n* = 49) groups. During the three-month intervention, both groups performed supervised sixty-minute training three times a week. All studied parameters were measured pre- and post-intervention period. In total, 85 women completed the study. Both training significantly decreased anthropometric parameters. Besides, endurance training decreased endothelial nitric oxide synthase, central aortic systolic pressure, pulse wave velocity, glutathione (GSH), total antioxidant status (TAS), interleukin (IL) 8, matrix metalloproteinase (MMP) 9, and tumor necrosis factor alpha, while endurance-strength training decreased MMP-2 concentrations, and increased IL-6, monocyte chemoattractant protein-1, and MMP-9 levels. We observed significant differences between groups for GSH, TAS, and MMP-9 levels. In summary, endurance and endurance-strength training did not differ in the impact on endothelial function and arterial stiffness. However, endurance training significantly depleted the antioxidant defense, simultaneously reducing MMP-9 levels. The study was retrospectively registered with the German Clinical Trials Register within the number DRKS00019832.

## 1. Introduction

It is well documented that obesity significantly increases the progression of atherosclerosis [1,2]. The main indicator of atherosclerosis is endothelial dysfunction, which is associated with an inappropriate vascular response, cell proliferation, thrombocyte activation, capillary permeability, and a pro-inflammatory phenotype [3]. Besides, endothelial dysfunction is also an important risk factor for cardiovascular diseases [4,5]. The mechanism linking excessive body weight and endothelial dysfunction has not been fully elucidated. However, obesity may disturb the production of pro-inflammatory cytokines and adipokines in adipose tissue. Indeed, pro-inflammatory markers are frequently increased in obese subjects. Simultaneously, anti-inflammatory parameters are reduced, which causes low-grade chronic inflammation, adipocyte dysfunction and endoplasmic reticulum stress and, in consequence, leads to endothelial dysfunction [6]. Interestingly, in obese subjects, significant sex differences in endothelial function were observed [7,8]. In particular, it seems that excessive body weight is associated with more prominent impaired resistance arteriolar endothelial function in women compared to men [9]. However, the rate of endothelial function decline with age may be greater in men than in women. It has been shown that brachial artery flow-mediated dilation (FMD) may be maintained until about 40 years of age in men, but in women, endothelial functions start to decline during the early menopause stage (around 50 years of age) [10,11,12]. Invasive (intra-arterial infusions of vasoacting agent) and noninvasive techniques (e.g., FMD method, pulse wave analysis (PWA), plethysmography method, or laser Doppler flowmetry) can assess endothelial function. Moreover, the vascular function can be evaluated by measuring the serum markers of endothelial dysfunction, including cell adhesion molecules, monocyte chemotactic protein 1 (MCP-1), vascular endothelial growth factor (VEGF) and inflammatory markers [13,14].

Several studies have shown that regular exercise training may reduce the risk of atherosclerosis by improving endothelial function. The positive influence of exercises on endothelial function includes stimulating the production of substances beneficial for its protection, such as superoxide dismutase (SOD) or nitric oxide (NO) [15,16]. Regular training also decreases vessel inflammation [17], reduces oxidative stress [18], increases microcirculation efficiency [19], and improves vascular formation [20]. Regular training also reduces body weight, decreases blood pressure (BP), improves lipid and carbohydrate homeostasis, as well as decreases thrombosis [21]. It is well known that exercises also decrease the risk of death from cardiovascular disease [22,23].

Currently, endurance training (also known as aerobic training) is one of the most recommended types of training for obese subjects. Moreover, it is suggested that the inclusion of a strength component into endurance training might intensify the beneficial effect of physical activity. However, the results of studies assessing the effect of different types of exercises of similar volume on endothelial functions are inconclusive [24,25,26,27,28]. Furthermore, previous studies focused on assessing the changes in FMD [24,26,27] and only few studies assessed endothelial markers, such as adhesion molecules [25,28]. Besides, it is suggested that the impact of exercises might differ in men and women, but the results of studies are varied [20,29,30,31]. Black et al. [29] observed that 24-weeks of training improved FMD and compound vascular function responses only in older females, which was not found in older males. Green et al. [30] also reported the sex-specific effect of training on popliteal and brachial arterial wall thickness, lumen diameters, as well as wall-to-lumen ratios. However, Pierce et al. [31] noticed improvements in FMD after eight weeks of endurance training only in elderly men, while in postmenopausal women no effect was found. There is also limited available data that compare the effect of endurance and endurance-strength training on endothelial markers, such as adhesion molecules, endothelial nitric oxide synthase (eNOS), metalloproteinases, VEGF, and others in obese women without serious comorbidities. In our pilot study, we did not observe any differences in changes in endothelial function parameters between applied training programs [25]. Therefore, we aimed to assess the effect of endurance and endurance-strength training on endothelial function in abdominally obese women in larger groups of obese women. The effects of both types of training on anthropometric parameters, arterial stiffness, antioxidant status, and inflammatory markers were also analyzed. We hypothesized that endurance and endurance-strength training do not differ in effect exerted on endothelial markers, anthropometric parameters, arterial stiffness, antioxidant status, and inflammatory markers. Since in our pilot study, we found a significant decrease of bone mineral density (BMD) at the lumbar spine after 12-weeks of training, we modified the endurance component of the exercise program and included in endurance training cycling with a load (standing cycling on command). Besides, we narrowed the age range (50–60 years) of involved subjects to obtain a more homogeneous population. As mentioned earlier, endothelial functions steep decline in women aged 50–60 years, which is associated with the loss of ovarian function and estrogen level reduction [10,11,12]. Therefore, we can assume that training intervention in this age group could prevent further deterioration of endothelial function.

## 2. Materials and Methods

### 2.1. Study Design and Ethical Issues

The present study was designed as a prospective parallel randomized trial and was performed in accordance with the guidelines of the Declaration of Helsinki. The study protocol was approved by the Poznan University of Medical Sciences Ethics Committee (References 219/16 and 1155/18). The study was per the standards of CONSORT (see Table S1: CONSORT 2010 checklist) [32] and was registered with the German Clinical Trials Register, registration number DRKS00019832 [33].

### 2.2. Study Population

Abdominally obese adult women were recruited and studied at the Poznan University of Medical Sciences (Poland). The assessment of suitability for study entry was performed by a medical doctor and consisted of medical history, medical examination, and available additional tests, including electrocardiography.

The inclusion criteria were as follows: age 50–60 years, obesity (body mass index (BMI) ≥ 30 kg/m^2^), waist circumference > 80 cm, percentage of fat mass ≥ 32% (the American Council on Exercise recommendation [34]) and no change in body weight in the month before the intervention (allowable deviation ±1 kg).

The exclusion criteria included: previously diagnosed secondary obesity or hypertension, type 2 diabetes mellitus (prediabetes subjects were not excluded), cardiovascular diseases including ischemic heart disease, stroke or transient ischemic attack, cardiac failure, heart rhythm problems or conduction disorders, poorly controlled hypertension (mean systolic BP > 140 mmHg and/or mean diastolic BP > 90 mmHg) during the month preceding the study, necessity to modify antihypertensive therapy in the last three months, lipid disorders requiring the pharmacological therapy in the previous three months, malignancy, use of any dietary supplements within three months before the intervention, liver, kidney or thyroid diseases, acute or chronic inflammation within the respiratory, digestive or urogenital system or in the mouth, throat and sinuses, connective tissue disease or arthritis, infection history in the mouth, abuse of nicotine, alcohol or drugs, pregnancy or labor at enrolment or in the three months before enrolment, breast-feeding in the three months prior to enrolment, and other disorders that could prevent, limit or confound the efficacy of the study.

In total, 101 obese postmenopausal women were recruited for the study. The participants were informed in detail about the study and that participation was voluntary with the possibility of withdrawing at any time without providing reasons. Written informed consent was obtained from all subjects. Participants were randomly divided into two groups: endurance training (*n* = 52) and endurance-strength training (*n* = 49). Table 1 summarizes the baseline characteristics of the study population. At baseline, there were no statistically significant differences between groups for analyzed parameters.

### 2.3. Intervention

Both groups performed three months of physical training, which varied only in the nature of the effort but had a comparable summary exercise volume. Aside from the training, all subjects were instructed to maintain their usual physical activity and diet. Pre- and post-intervention, anthropometric parameters, markers of endothelial dysfunction, antioxidant status, inflammatory process, as well as arterial stiffness were assessed in both groups, as described previously [35]. The intervention included a physical exercise program which consisted of three sessions of training per week (on Monday, Wednesday, and Friday), with a total of 36 training sessions for each group. The subjects should complete 80% of training (29 training sessions) to be included in the analysis. The exercise routine was supervised by a professional fitness instructor, medical doctor, and study team members. All sessions were performed in the afternoon in a professional training room at the Sports Club City Zen, Poznan, Poland. The endurance group was trained on cycle ergometers (Schwinn Evolution, Schwinn Bicycle Company, Boulder, CO, USA) and the training consisted of five minutes of warm-up, 45 min of endurance training with a load at an intensity between 50–70% of maximum heart rate (HR max), five minutes of cycling without load, and five minutes of stretching and breathing exercises. Importantly, the intensity of endurance training in both groups was individually selected for each participant and did not change during the intervention period. Similarly, the endurance-strength training session (also known as mixed or combined training) included five minutes of warm-up, resistant and endurance exercises, cycling without load, and cool down stretches. The resistant component included 20 min of strength exercises with a barbell and a gymnastic ball at 50–60% of one-repetition maximum. Mondays and Fridays consisted of upper and lower limb exercises with a barbell, while training on Wednesdays was with a gymnastic ball. Each session included the same number of exercises and repetitions depending on their muscle strength; the goal was 16 barbell bicep curls and 30 barbell squats. A 10–15 s break was taken between the series of strength exercises, during which subjects performed isometric exercises. Directly after the strength training, women performed 25 min of endurance training with a load on cycle ergometers (Schwinn Evolution, Schwinn Bicycle Company, Boulder, CO, USA) of intensity between 50–70% of HR max, five minutes of cycling without load, and five minutes of low-intensity cool down stretching and breathing exercises. HR during training was monitored with a Suunto Fitness Solution device (Suunto, Vantaa, Finland). The adherence to the intervention was assessed based on the attendance to the training sessions. An attendance list was kept during each training session to calculate the percentage of completed training sessions. Our previous pilot study also assessed the effect of 12-week endurance and endurance-strength training programs on anthropometric parameters and selected biochemical parameters. However, the pilot study included a small number of subjects aged 28–62 [25]. Due to the negative effect of training on bone health observed in our pilot trial, we slightly modified endurance training, including cycling with a load.

### 2.4. Study Outcomes

The primary outcomes were changes in endothelial functions in the course of the intervention. Secondary outcomes were changes in anthropometric parameters, arterial stiffness, antioxidant status markers, and inflammatory parameters. Methods used to measure the outcomes did not differ between groups.

### 2.5. Graded Exercise Test (GXT)

Physical capacity was measured using the GXT, which was performed at the beginning of the intervention on an electronically braked cycle ergometer (Kettler DX1 Pro, Ense-Parsit, Germany). The test started at a work rate of 25 W (60 revolutions/min) and the rate was systematically increased by 25 W every two minutes until the participants were not able to maintain the required pedal cadence. The duration depended on the subjects’ age and aerobic fitness status, and lasted approximately 4–14.5 min. The assessments were performed between 8:00 a.m. and 12:00 a.m. in an air-conditioned laboratory two hours after consuming a light breakfast. Oxygen intake (VO_2_) and carbon dioxide output (VCO_2_) min ventilation, and HR were measured using an automated system (Oxycon Mobile; Viasys Healthcare, Höchberg, Germany). Before each test, the calibration was performed according to the manufacturer’s instructions. Temperature, humidity, and barometric pressure were monitored. VO_2_ peak was measured as the highest value of VO_2_ obtained during the highest exercise intensity, while HR peak was defined as the highest value achieved during the GXT. Time to exhaustion was the time remaining before the subjects were not able to maintain the given and maximal work rates defined as the highest work rate achieved during the test. The V-slope method was used to determine the ventilatory threshold (VT). The method analyzes the behavior of VCO_2_ as a function of VO_2_ during GXT with a consequent increase in VCO_2_. This results in a transition in the relationship between VCO_2_ and VO_2_. The Viasys Healthcare (Conshohocken, PA, USA) software was used. VT was also measured using the ventilatory equivalent method. The point at which the equivalent for O_2_ increased without a concomitant rise in the equivalent for CO_2_ was detected. The VT was expressed as the work rate and HR.

### 2.6. Blood Pressure

The BP was measured according to the European Society of Hypertension Guidelines [36]. The measurements were performed during the recruitment visit as well as before the GXT. The BP at the VT was also measured during the GXT. Resting HR was monitored using a stethoscope to auscultate the heart. The BP and HR measurements were taken during fasting in the morning hours in a sitting position with the legs uncrossed, and with the back and arm supported.

### 2.7. Anthropometry Parameters and Body Composition

The anthropometric assessment comprised body height, body weight, and waist circumferences. During the morning measurements, women were fasting and were barefoot wearing light clothing. Body weight and height were measured by a calibrated electronic scale with a stadiometer (model WPT 100/200 OW from RadWag, Radom, Poland). Waist circumference was determined at the horizontal plane, midway between the lowest rib and iliac crest using a standard measuring tape (Seca, Hamburg, Germany). Based on the anthropometric measurement, BMI was calculated by dividing body weight (kg) by the square of body height (m^2^). In this study, obesity was recognized according to the World Health Organization criteria [37]. Waist circumference was categorized according to cut-off points defined by the International Diabetes Federation [38]. Additionally, body composition was assessed during the recruitment process using the bioimpedance analysis with the InBody 370 analyzer (InBody Co. Ltd., Seoul, South Korea). Body composition was evaluated to check if participants fulfill the inclusion criteria. We used 32% of body fat as a criterium for diagnosing obesity in women, which is in line with the American Council on Exercise recommendation [34].

### 2.8. Arterial Stiffness

Arterial stiffness was assessed by the SphygmoCor system (SphygmoCor, AtCor, Sydney, Australia). BP and arterial stiffness parameters were measured in the supine position after five minutes of rest at the left side of the body. Cuffs were chosen individually for the subjects. To measure pulse wave velocity (PWV), the location of the strongest carotid and femoral pulse were found and the distance between these two was measured. The waveform was analyzed by the SphygmoCor probe placed on the pre-determined carotid pulse location. The HR and the pulse transit time from the carotid to the femoral artery were also monitored. To measure PWA a cuff was placed at the level of the brachial artery of the subject’s arm. The following parameters were assessed during the PWA: Central aortic systolic pressure (SP), central aortic pulse pressure (PP), augmentation index (AIx), and augmentation pressure (AP).

### 2.9. Biochemical Measurements

Baseline and seven-day after the intervention period fasting blood samples were collected from all study participants. Markers of 1/endothelial dysfunction (A/asymmetric dimethylarginine—ADMA; SunRed Human ADMA ELISA Kit, Shanghai, China, B/eNOS; MyBioSource Human Endothelial Nitric Oxide Synthase ELISA kit, San Diego, CA, USA, C/homocysteine—HCY; Axis Homocysteine EIA kit, Dundee, United Kingdom, D/plasminogen activator inhibitor-1—PAI-1; Human Total Serpin E1/PAI-1 Quantikine ELISA, R&D Systems a Biotechne brand, Minneapolis, MN, USA, E/soluble vascular cell adhesion molecule-1—sVCAM-1; sVCAM-1 (human) kit, DRG Instruments GmbH, Marburg, Germany; F/VEGF; Human VEGF, (Quantikine ELISA, R&D Systems a Biotechne brand, Minneapolis, MN, USA), 2/antioxidant status (A/glutathione—GSH; Human Reduced GSH ELISA Kit, MyBiosource, San Diego, CA, USA, B/superoxide dismutase—SOD; SOD Assay Kit, Cayman Chemical, Ann Arbor, MI, USA, C/total antioxidant status—TAS; Human TAS ELISA kit, Qayee-bio, Shanghai, China) and 3/inflammatory status (A/high-sensitivity interleukin-6—hs-IL-6; Human IL-6 Immunoassay, Quantikine HS ELISA, R&D Systems a Biotechne brand, Minneapolis, MN, USA, B/high-sensitivity interleukin-8—hs-IL-8; Human CXCL8/IL-8 Immunoassay, Quantikine HS ELISA, R&D Systems a Biotechne brand, Minneapolis, MN, USA, C/MCP-1; MCP-1 human ELISA, DRG Instruments GmbH, Marburg, Germany, D/matrix metalloproteinase-2—MMP-2; Total MMP-2 Immunoassay, Quantikine ELISA, R&D Systems a Biotechne brand, Minneapolis, MN, USA, E/matrix metalloproteinase-9—MMP-9; Human MMP-9 Immunoassay, Quantikine ELISA, R&D Systems a Biotechne brand, Minneapolis, MN, USA, F/tumor necrosis factor alpha—TNF-α; human tumor necrosis factor alfa, ELISA kit, Qayee-bio, Shanghai, China) were measured by standard clinical chemical assays at the Laboratory of the Department of Pediatric Gastroenterology and Metabolic Diseases, Poznan University of Medical Sciences (Poznan, Poland). Serum high-sensitivity C reactive protein (hs-CRP) concentrations were measured by an immunoturbidimetric method at ALAB Laboratory (Poznan, Poland). Besides, serum nitrite (NO_2_) and nitrate (NO_3_) concentrations were measured at the Department of Pediatric Gastroenterology and Metabolic Diseases, Poznan University of Medical Sciences (Poznan, Poland) according to methods described by Tsikas [39].

### 2.10. Randomization

Subjects were randomly divided into two groups by an independent researcher using a randomization list (allocation ratio 1:1). Blocked randomization was conducted with the stratification according to age, body weight, BMI, and waist circumference. The allocation sequence concealed until participants were enrolled and assigned to interventions. After the study was started, there was no blinding to study participants, health professionals, and other research staff involved in the trial; outcome assessors and study team members who prepared the database and performed the statistical analysis were blinded.

### 2.11. Sample Size Calculation

The G*Power 3.1.9.2 software (University of Kiel, Kiel, Germany) was used to calculate the minimum sample size. The calculation based on the change in endothelial function parameter (eNOS) was previously reported in our pilot study [25]. To obtain a power of 80% (α = 0.05, β = 0.2), around 40 subjects per group should be required; however, assuming that 20% of subjects may withdraw from the study, a minimum of 48 subjects should be included in each group.

### 2.12. Statistical Analysis

STATISTICA 13.0 (TIBCO Software Inc., Palo Alto, CA, USA) software was used for statistical analyses. A two-sided *p*-value of less than 0.05 was regarded as statistically significant. The assumption of normality was verified using the Shapiro–Wilk test. Data were expressed as medians and the interquartile interval (Q1–Q3), as well as means and standard deviations (SDs) with the 95% confidence interval of means (95% CI), both for the direct measurement values and the changes between pre- and post-intervention values (Δ value at week 12). Comparisons between unpaired data were performed using the Mann–Whitney U test, whereas dependent variables (pre- and post-intervention) were compared using the Wilcoxon rank-sum test. Spearman correlation between baseline BMI and changes in endothelial parameters, arterial stiffness, antioxidant markers, and inflammatory parameters were performed in the total population and separately in the endurance group and the endurance-strength group to assess how obesity may modulate the response to exercise training. Moreover, we also correlated the changes in BMI with the changes in other outcomes.

## 3. Results

### 3.1. Participants Flow

**Figure 1:**
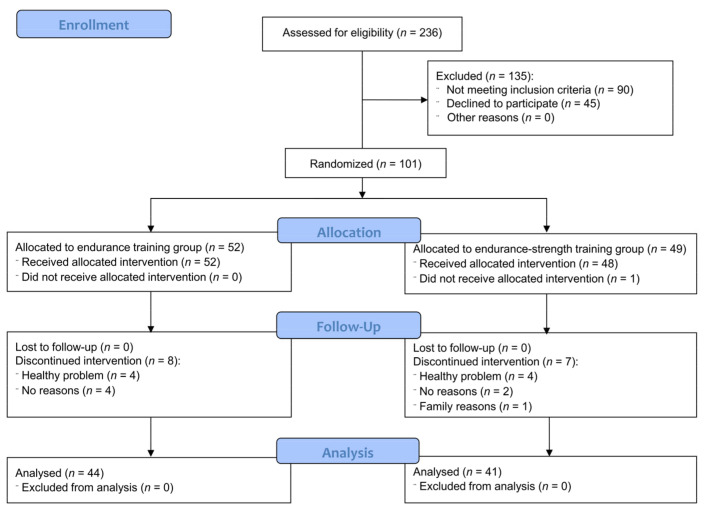
CONSORT 2010 flow diagram.

Subjects were recruited to the study between January and August 2016, while the intervention period was divided into two parts: the first started in April 2016 and finished in June 2016 (spring session) and the second respectively in September and November 2016 (autumn session). Forty-eight women participated in the spring session and 53 women took part in the autumn session. Figure 1 presents participant flow. In total, 236 subjects were assessed for eligibility. Out of them, 135 subjects were excluded (90 subjects did not meet the inclusion criteria and 45 subjects declined to participate). Eventually, 101 subjects were randomly divided into the endurance training group (*n* = 52) and the endurance-strength training group (*n* = 49). All subjects from the endurance group and 48 subjects from the mixed group received allocated intervention. Fifteen subjects (eight from the endurance group and seven from the endurance-strength group) discontinued the intervention (eight due to health reasons, six dropped out of the study without explanation, and one due to family reasons). No significant side effects were reported. Five persons reported joint pain, in two subjects high blood pressure was observed after the training session, one subject had muscle pain and in one participant swelling occurred.

### 3.2. Effects of Endurance and Endurance-Strength Training on Anthropometric Parameters, Endothelial Function, Arterial Stiffness, Antioxidant Status and Inflammatory Markers

In total, 85 postmenopausal women (84.2%; 44 from endurance training and 41 from endurance-strength training groups) completed the study. Pre- and post-intervention values of each outcome are presented in Table 2. There were no differences in baseline values between subjects from both groups who were included (see Table 1) and completed (see Table 2) the intervention period. The mean adherence (percentage of completed training sessions) in the subjects who completed the study was 91%, with no differences between groups. Endurance training significantly decreased body weight (*p* < 0.001), BMI (*p* < 0.001), waist circumference (*p* < 0.001), PWA SP (*p* < 0.001), PWV (*p* < 0.001), eNOS (*p* = 0.01), GSH (*p* = 0.003) and TAS levels (*p* < 0.001), as well as serum hs-IL-8 (*p* = 0.009), MMP-9 (*p* = 0.04), and TNF-α (*p* = 0.003) concentrations, while endurance-strength training significantly increased serum hs-IL-6 (*p* = 0.03), MCP-1 (*p* = 0.007) and MMP-9 (*p* = 0.05) concentrations, and decreased body weight (*p* = 0.03), BMI (*p =* 0.02), waist circumference (*p* < 0.001) and MMP-2 levels (*p* = 0.003).

### 3.3. Comparison of the Effect of Endurance and Endurance-Strength Training on Anthropometric Parameters, Endothelial Function, Arterial Stiffness, Antioxidant Status and Inflammatory Markers

Comparison of the effect of endurance and endurance-strength training on anthropometric parameters, endothelial function, arterial stiffness, antioxidant status, and inflammatory markers is presented in Table 3. Endurance and endurance-strength training did not differ in effect on endothelial function markers, arterial stiffness, and oxidative stress parameter. However, there were significant differences between the effect of intervention program on GSH (mean (95% CI): −2.70 (−4.54–−0.85) vs. 1.85 (−0.96–4.65) µmol/L, *p* = 0.003), TAS (mean (95% CI): −74 (−131–−17) vs. −29 (−112–55) ng/mL, *p* = 0.002) and MMP-9 levels (mean (95% CI): −89.4 (−167.9–−10.8) vs. 103.2 (−0.7–207.2) ng/mL, *p* = 0.003).

### 3.4. Correlation between BMI and Changes in the Main Outcomes

In total population, baseline BMI negatively correlated with changes in SOD (r = −0.2543, *p* = 0.02), hs-CRP (r = −0.2755, *p* = 0.01), VEGF (r = −0.2385, *p* = 0.03). A similar association between BMI and changes in hs-CRP (r = −0.3030, *p* = 0.05) as well as VEGF (r = −0.4019, *p* = 0.007) was noted in the endurance group. However, in this group, we also found a negative correlation between baseline BMI and changes in MMP-9 (r = −0.3283, *p* = 0.03). On the other hand, in the endurance-strength group, pre-intervention BMI negatively correlated with changes in SOD (r = −0.4195, *p* = 0.006) and PWA AP (r = −0.3974, *p* = 0.01), while a positive correlation was observed between baseline BMI and PAI-1 (r = 0.3276, *p* = 0.04). Besides, changes in BMI significantly correlated with changes in MMP-9 in the total population (r = 0.3381, *p* = 0.002) and mixed group (r = 0.5108, *p* < 0.001). In the endurance-strength group, a positive correlation between changes in BMI and VEGF was found (r = 0.3577, *p* = 0.02). 

## 4. Discussion

Key findings of this study include significant differences between the effects of endurance and endurance-strength training on selected oxidative stress markers and inflammatory parameters (GSH, TAS, and MMP-9 concentrations). However, we did not notice any differences regarding endothelial markers and arterial stiffness. What’s important, in both groups significant reductions of selected anthropometric parameters were found. To our knowledge, this is the first adequately sized study that compared the effect of endurance and endurance-strength training on endothelial parameters in women with abdominal obesity without serious comorbidities.

It is well known that physical exercises have a beneficial effect on anthropometric parameters [40]. In the present study, we observed that endurance and endurance-strength training were effective in the reduction of body weight, BMI, and waist circumference. However, no significant differences between the effect of both types of training on anthropometric parameters were observed. Similar results have been demonstrated in several [25,40,41,42], albeit not all [43,44], studies. Our previous pilot study showed that both endurance and combined training had a positive effect on anthropometric parameters. However, previously we did not compare changes in anthropometric parameters between both types on training [25,40]. Sanal et al. [41] also noted that 12-weeks of endurance and endurance-strength training significantly reduced body weight, BMI, waist and hip circumferences in overweight and obese subjects. Besides, there were no significant differences in these measurements between groups. AbouAssi et al. [42] also noted that after eight months of intervention, both endurance and endurance-strength training (which required twice as much time as either endurance training alone) cause a similar decrease in body weight in moderate dyslipidemia and overweight subjects. However, Ho et al. [43] observed that mixed training compared to the same volume of endurance or strength training was significantly more effective in the reduction of body weight after eight and 12-weeks of intervention in overweight and obese subjects. Schwingshackl et al. [44] in a meta-analysis also showed that endurance-strength training was more effective in improving anthropometric parameters. Furthermore, it should be highlighted that the effect of training programs on anthropometric parameters may vary according to the sex of the participants. Donnelly et al. [45] showed that exercises induced a more pronounced reduction in body weight in men than women and reported that endurance training prevented weight gain in women but produced weight reduction in men. The difference in body composition between men and women could partly explain these results [46].

Previous studies have also shown that exercises may decrease cardiovascular risk by preserving endothelial function [47,48,49]. Several mechanisms have been proposed to explain the beneficial effect of exercise training on endothelial parameters. It was documented that regular training augments blood flow and laminar shear stress, which increases the production and bioavailability of NO. The positive outcome of training sessions on endothelial function can also include synthesis of molecular mediators, changes in the neurohormonal production, and oxidant stress or antioxidant status. Moreover, physical training may also prompt angiogenesis-related systemic molecular pathways, affect chronic anti-inflammatory action and, therefore, impact endothelial function. However, its benefit depends on the intensity of training. It has been shown that only regular moderate-intensity training has a positive impact on the antioxidant status and maintains endothelial function [50]. It is also supposed that the effect of exercises on endothelial function may also depend on the type of training. However, the results of studies assessed the impact of different type of physical intervention on endothelial function are inconclusive [24,25,26,27,28]. Moreover, previous studies assessed endothelial function mostly by changes in FMD [24,26,27,51,52,53,54] and only a few studies evaluated endothelial functions using biochemical parameters, e.g., ADMA, eNOS, PAI-1, sVCAM, VEGF [25,28]. However, it should be mentioned that no direct correlation between endothelial dysfunction biomarkers and changes in endothelial function assessed by FMD has been documented to date. Moreover, vascular biomarkers might not directly reflect the local tissue environment [55,56,57]. Schjerve et al. [26] observed that both endurance and strength training significantly improve endothelial function measured as FMD in obese subjects after 12 weeks of intervention, but high-intensity aerobic training was more effective in the improvement of endothelial function compared with strength training and moderate-intensity groups. Besides, Olson et al. [52] demonstrated that one year of strength training improved peak FMD in overweight women and the changes were independent of anthropometric parameters, BP, lipid profile, glucose, and insulin levels. Furthermore, a recent systematic review showed that aerobic exercises decreased the levels of adhesion molecules. However, the effect of a training program varies depending on the type and duration of the exercises [58]. In the present study, no effect of endurance-strength training on endothelial function parameters was observed, but endurance training significantly reduced eNOS levels. Besides, there were no significant differences between the impact of both types of exercises on endothelial markers. Contrary, our pilot study showed that endurance training significantly increased eNOS levels; however, both endurance and endurance-strength training did not affect VEGF concentrations [25]. Furthermore, Thomson et al. [28] compared the effect of diet, diet combined with the similar volume of aerobic or aerobic-resistance exercises on endothelial function in overweight or obese women with polycystic ovary syndrome and showed a decrease of PAI-1 and sVCAM-1 levels with weight loss in all groups and no change in ADMA levels. However, no differences between groups were found. On the other hand, Østergard et al. [53] reported no changes in endothelial function measured by FMD after 10-weeks of aerobic training in obese subjects with type 2 diabetes. Based on these results, it has been suggested that only a long-term exercise program associated with a higher frequency of exercises might lead to improved vascular endothelial function. Nevertheless, the gender may affect the findings considering that women have more prominent impaired resistance arteriolar endothelial function compared to men [9].

We also did not observe differences between the effect of endurance and endurance-strength training on arterial stiffness. However, endurance training significantly decreased PWA SP and PWV. Our results are in line with the previous meta-analysis, which showed that the effect of aerobic training on arterial stiffness did not differ compared with combined training. However, it was suggested that the impact of exercise training on arterial stiffness could be influenced by the health status of the study subjects [59]. Daily physical activity may also have an impact on arterial stiffness. It has been shown that sedentary activity is associated with increased arterial stiffness [60]. However, in our study, we did not assess usual daily physical activity and, therefore, we could not analyze the impact of daily physical activity on the obtained findings. The hormonal status might also affect arterial stiffness. It is well known that menopause may increase the arterial stiffness, which is associated with estrogen deficiency. However, the positive effect of training on arterial stiffness has been reported in both pre- and post-menopausal women [61,62,63].

Several studies have shown that regular exercises might have a beneficial effect on antioxidant status, alleviating the negative effects caused by free radicals [64,65], with acute and intense physical exercise causing depletion of antioxidant reserves and induction of oxidative stress. As a result of acute training, increased lipid peroxidation, O_2_ consumption in the muscles, superoxide anion generation, and impairment of the antioxidant system were observed [66]. It was also shown that the intensification of oxidative stress depends not only on the intensity of training but also on the duration of exercises and the current level of fitness [67]. However, to date, only a few studies compared the effect of various types of training on antioxidant parameters [25,68,69,70,71]. Azizbeigi et al. [69] reported that eight-week endurance, strength, and mixed training (both programs performed alternatively) significantly reduced oxidative stress and increased antioxidant capacity in men with no active lifestyle, with increased activity of glutathione peroxidase in erythrocytes in endurance and mixed training groups, but not in the strength training group. Moreover, an increase in SOD activity was observed in all groups, while TAS increased in endurance and endurance-strength groups, but not in the strength training group. Nevertheless, the differences between the individual groups were not statistically significant. De Oliveira et al. [68] compared the effect of aerobic, strength, and mixed training (including endurance training performed at a similar intensity and half of the volume of that in the aerobic group) on the oxidative stress parameters in subjects with type 2 diabetes and showed an increase in SOD activity after 12-week aerobic training, while strength training and combined training did not affect this parameter. There were no changes in glutathione peroxidase activity and TAS levels in any of the groups. In contrast, García-López et al. [70] did not show any effect of 21-week endurance and strength training on SOD and glutathione peroxidase activities in middle-aged men. However, Linke et al. [71] observed a decrease in SOD and glutathione peroxidase activity in skeletal muscle after six-month endurance workouts in subjects with chronic heart failure. In our pilot study, we observed a decrease in thiobarbituric acid reactive substance concentrations in the endurance group, but TAS did not change in both groups [25]. Herein, we reported significant differences between groups in the effect on GSH and TAS levels. We found that endurance training significantly depleted antioxidant reserves compared to mixed training. The negative effect of endurance training on antioxidant status may be related to the hormonal status of women. Indeed, menopause is a risk factor for oxidative stress, considering that a decrease in antioxidant defense is frequently observed in postmenopausal women [72].

The effect of exercise training on inflammatory markers has been discussed recently in several studies, but the data are conflicting [25,73,74,75]. Ratajczak et al. [25] found a decrease in CRP levels in the combined training group but not in the endurance training group. Giannopoulou et al. [73] observed that 14-week aerobic exercises decreased CRP and IL-6 levels but did not change TNF-α concentrations in obese postmenopausal women with type 2 diabetes, while Jorge et al. [74] noted a decrease in CRP levels in aerobic, resistance, and combined groups (all training on the same volume), and no statistically significant increase in TNF-α and IL-6 levels after 12-week resistance training in subjects with type 2 diabetes mellitus. Similarly, Libardi et al. [75] showed that 16-week resistance, endurance, and concurrent training performing three days per week and lasting about 60 min, did not affect IL-6, TNF-α, and CRP levels in healthy middle-aged men. Recent studies have also provided conflicting results regarding the effect of exercises on MCP-1 levels [76,77,78]. Trøseid et al. [76] observed a statistically significant reduction of MCP-1 levels in the combined training group with metabolic syndrome as compared to the non-exercise subjects. It was also demonstrated that endurance exercises decrease MCP-1 levels [77]. On the other hand, Suzuki et al. [78] observed that MCP-1 concentrations increased after short-duration exercises. Previously, it was also reported that alterations in MMP-2 and MMP-9 levels might reflect the impact of training on the inflammation [79]. However, at present, the effect of different training programs on MMP-2 and MMP-9 levels is still unknown. According to recent studies, the impact of training on MMPs levels may depend on the type and length of training. Resistance training lasting 5–12 weeks may increase MMPs levels [80,81], whereas an acute bout of resistance training decreases MMP-2 and MMP-9 concentrations [82]. Long-term endurance exercises, lasting up to 12 weeks, have reduced MMP-2 and MMP-9 levels [83], while MMPs increased following acute bouts of exercise [84]. A recent systematic review also confirmed that chronic endurance exercises reduce MMPs concentrations and have a cardioprotective effect, while strength training displayed conflicting results [79]. Our findings showed that endurance and endurance-strength training programs exerted different effects on inflammatory markers. Endurance training reduced serum hs-IL-8, MMP-9, and TNF-α concentrations, while endurance-strength training increasing hs-IL-6, MCP-1, MMP-9 levels, simultaneously reducing MMP-2 levels. However, a significant difference between both types of training was observed only for MMP-9 levels. Differences between results of subsequent studies could be partly explained by gender. Women have higher inflammatory markers levels compared with men, which may be due to a higher proportion of fat [85,86].

Limitations of this study included a lack of separate strength and control groups which limit interpretation of the obtained results. We also did not measure FMD, whereas the assessment of FMD in the conjunction with endothelial markers might provide better insight into the effect of both types of training on endothelial functions. Lack of cell and tissue-specific measurements is another limitation of the study as it is not clear how some parameters measured in blood align with the status of the parameters at the level of the endothelium. Another limitation of the study is that we did not measure oxidative stress parameters, while several markers of antioxidant status were assessed. Furthermore, we did not list in the inclusion criteria subjects’ hormonal status and using oral contraceptives or hormone replacement therapy in the exclusion criteria. However, eventually, all subjects who were included in the study were postmenopausal women. Moreover, the participants’ hormonal status was assessed based on the subjects’ declarations, and we did not measure sex-hormone levels in the blood. Additionally, daily physical activity was not assessed. However, the subjects were instructed not to change their usual physical activity during the intervention period. Besides, this study was conducted on obese women without major comorbidities. However, the coexistence of serious complications is frequently observed in obese subjects aged 50–60 years, so including obese subjects without obesity-related complications might explain no changes in some biochemical parameters observed in this study. It is of note, that results obtained in obese subjects with serious comorbidities might be quite different, so our findings cannot be generalized to the general population of obese subjects. Besides, our investigation focused on women. Therefore, it is unknown if the training intervention would result in similar changes in men. Moreover, it was reported that the effect of exercise training might differ in subjects of different ages. Indeed, training efficiency decreases and O_2_ cost of exercise increases in older subjects, which contributes to a reduced training capacity [87].

Our comprehensive evaluation of the study population and the inclusion of a large variety of biochemical parameters provides accurate prediction of the endothelial functions in women with abdominal obesity. Besides, we used very strict inclusion and exclusion criteria, which eliminated the influence of disrupting factors. Moreover, as was mention earlier, to the best of our knowledge, this is one of the first randomized trials that compared the effect of endurance and endurance-strength training on endothelial parameters in women with abdominal obesity. Our previous pilot study also assessed the impact of 12-week endurance training and combined training on selected endothelial (eNOS, VEGF), antioxidant (TAS, thiobarbituric acid reactive substances), and inflammatory (CRP) parameters. However, the pilot study included a small number of subjects (*n =* 39) aged 28–62 [25], while in this study we included a large cohort of women aged 50–60 years. Besides, due to the negative effect training program on the bone health observed in the pilot study (decrease of BMD at the lumbar spine; we decided to modified endurance exercises. Therefore, in this study, in the endurance program, we included cycling with a load (standing cycling on command).

## 5. Conclusions

In conclusion, in our population of middle-aged, postmenopausal women with abdominal obesity, a three-month endurance and endurance-strength training did not differ in the effect on anthropometric parameters, endothelial function, and arterial stiffness. However, endurance training significantly depleted the antioxidant defense, simultaneously reducing MMP-9 levels. Further studies are needed to investigate the potential mechanism responsible for changes in oxidative markers after endurance training.

## Figures and Tables

**Table 1:** Baseline characteristics of study population (*n* = 101).

	Endurance Training (*n* = 52)		Endurance-Strength Training (*n* = 49)		*p*
	Mean ± SD (95% CI)	Median (Q1–Q3)	Mean ± SD (95% CI)	Median (Q1–Q3)	
Anthropometric parameter					
Age (years)	55 ± 7 (53–57)	55 (50–60)	55 ± 7 (53–58)	54 (50–60)	0.84
Weight (kg)	96.0 ± 15.1 (91.7–100.2)	93.4 (84.9–104.9)	93.2 ± 13.9 (89.2–97.2)	91.0 (82.4–101.8)	0.41
BMI (kg/m^2^)	35.87 ± 4.43 (34.63–37.10)	35.64 (32.07–38.00)	35.98 ± 5.10 (34.52–37.45)	35.42 (31.79–39.10)	0.86
Waist circumference (cm)	110 ± 10 (107–113)	109 (103–114)	110 ± 10 (107–113)	108 (103–117)	1.00
Endothelial function parameters					
ADMA (nmol/mL)	1.66 ± 1.47 (1.25–2.07)	0.98 (0.44–2.90)	1.38 ± 1.35 (0.99–1.77)	0.59 (0.40–2.26)	0.25
eNOS (U/L)	29.19 ± 28.03 (21.39–37.00)	20.97 (14.17–32.73)	24.86 ± 23.18 (18.21–31.52)	18.76 (14.43–27.23)	0.75
HCY (µmmol/L)	10.86 ± 2.73 (10.10–11.62)	11.03 (9.12–12.46)	10.70 ± 2.87 (9.87–11.52)	10.21 (8.94–11.61)	0.26
NO_2_ (µmol/L)	1.42 ± 0.28 (1.34–1.39)	1.36 (1.19–1.66)	1.40 ± 0.30 (1.31–1.48)	1.38 (1.22–1.55)	0.66
NO_3_ (µmol/L)	36.69 ± 17.70 (31.76–41.61)	31.37 (24.59–43.69)	36.44 ± 16.04 (31.84–41.05)	31.98 (27.22–40.49)	0.58
PAI-1 (ng/mL)	118.9 ± 33.0 (109.7–128.1)	119.6 (91.5–134.7)	119.1 ± 40.5 (107.5–130.7)	115.0 (86.0–141.0)	0.97
sVCAM-1 (ng/mL)	808.95 ± 203.90 (752.19–865.72)	750.00 (685.50–872.45)	808.42 ± 257.65 (734.41–882.42)	764.70 (651.00–876.00)	0.92
VEGF (pg/mL)	485 ± 278 (408–563)	436 (266–601)	471 ± 258 (397.36–545.54)	431 (239–638)	0.80
Arterial stiffness					
PWA SP (mmHg)	129 ± 12 (126–133)	129 (120–138)	131 ± 17 (126–136)	130 (122–138)	0.88
PWA PP (mmHg)	42 ± 11 (39–45)	31 (37–47)	44 ± 14 (39–48)	42 (35–51)	0.76
PWA AIx	31 ± 14 (27–35)	30 (22–36)	30 ± 14 (26–34)	30 (19–39)	1.00
PWA AP (mmHg)	13 ± 6 (12–15)	13 (9–18)	134 ± 8 (11–20)	13 (7–19)	0.99
PWV (m/s)	7.1 ± 1.2 (6.8–7.5)	7.0 (6.3–7.9)	6.7 ± 1.2 (6.4–7.1)	6.5 (5.8–7.5)	0.08
Antioxidant status parameters					
GSH (µmol/L)	20.60 ± 25.97 (13.37–27.83)	13.22 (9.69–23.14)	24.83 ± 72.27 (4.01–45.64)	11.47 (6.69–20.54)	0.44
SOD (U/mL)	3.44 ± 2.03 (2.87–4.00)	3.48 (1.75–4.31)	3.12 ± 1.24 (2.76–3.47)	2.92 (2.13–4.19)	0.42
TAS (ng/mL)	674 ± 865 (433–915)	242 (184–675)	577 ± 809 (346–810)	195 (166–370)	0.35
Inflammatory markers					
hs-CRP (mg/L)	3.99 ± 3.97 (2.88–5.09)	2.95 (1.10–5.35)	4.16 ± 4.08 (2.99–5.33)	2.30 (1.70–4.80)	0.95
hs-IL-6 (pg/mL)	2.32 ± 2.07 (1.75–2.90)	1.85 (1.22–2.58)	2.05 ± 1.52 (1.61–2.48)	1.57 (1.33–2.31)	0.45
hs-IL-8 (pg/mL)	33.80 ± 26.17 (26.51–41.09)	23.52 (18.60–42.94)	31.61 ± 33.34 (22.03–41.18)	22.45 (13.01–34.27)	0.39
MCP-1 (pg/mL)	415.51 ± 189.20 (362.83–468.18)	402.55 (296.80–532.85)	416.42 ± 165.19 (368.97–463.87))	367.85 (298.05–495.35)	0.91
MMP-2 (ng/mL)	209.30 ± 48.38 (195.83–222.77)	203.37 (177.00–236.74)	207.60 ± 54.71 (191.89–223.32)	201.00 (176.54–231.72)	0.38
MMP-9 (ng/mL)	746.7 ± 292.9 (665.1–828.2)	736.3 (522.1–936.0)	767.3 ± 412.3 (648.9–885.7)	682.7 (467.6–841.5)	0.86
TNF-α (pg/mL)	43 ± 55 (27–58)	15 (11–46)	33 ± 46 (19–46)	12 (10–19)	0.30

ADMA—asymmetric dimethylarginine; BMI—body mass index; eNOS—endothelial nitric oxide synthase; GSH—glutathione; HCY—homocysteine; hs-CRP—high-sensitivity C reactive protein; hs-IL-6—high-sensitivity interleukin-6; hs-IL-8—high-sensitivity interleukin-8; MCP-1—monocyte chemotactic protein 1; MMP-2—matrix metalloproteinase-2; MMP-9—matrix metalloproteinase-9; NO_2_—nitrite; NO_3_—nitrate; PAI-1—plasminogen activator inhibitor-1; PWA AIx—augmentation index; PWA AP—augmentation pressure; PWA SP—central aortic systolic pressure; PWA PP—central aortic pulse pressure; PWV—pulse wave velocity; SD—standard deviation; SOD—superoxide dismutase; sVCAM-1—soluble vascular cell adhesion molecule-1; TAS—total antioxidant status; TNF-α—tumor necrosis factor alpha; VEGF—vascular endothelial growth factor; 95% CI—95% confidence interval.

**Table 2:** Effects of endurance and endurance-strength training on endothelial function, arterial stiffness, oxidative stress, antioxidant status, and inflammatory markers.

	Endurance Training (*n* = 44)				*p*	Endurance-Strength Training (*n* = 41)				*p*
	Pre-Intervention		Post-Intervention			Pre-Intervention		Post-Intervention		
	Mean ± SD (95% CI)	Median (Q1–Q3)	Mean ± SD (95% CI)	Median (Q1–Q3)		Mean ± SD (95% CI)	Median (Q1–Q3)	Mean ± SD (95% CI)	Median (Q1–Q3)	
Anthropometric parameter										
Weight (kg)	94.6 ± 14.8 (90.1–99.1)	90.4 (83.6–101.7)	93.4 ± 14.5 (89.0–97.8)	90.1 (82.1–99.3)	<0.001	93.3 ± 13.4 (89.1–97.5)	93.1 (82.4–101.8)	92.1 ± 13.9 (87.8–96.5)	90.7 (82.1–102.7)	0.03
BMI (kg/m^2^)	35.45 ± 4.51 (34.07–36.82)	34.41 (31.63–37.46)	35.00 ± 4.32 (33.69–36.32)	34.31 (31.30–37.49)	<0.001	35.72 ± 4.63 (34.25–37.18)	35.42 (31.60–39.06)	35.26 ± 4.74 (33.76–36.76)	34.35 (31.52–38.83)	0.02
Waist circumference (cm)	109 ± 10 (106–112)	109 (103–113)	106 ± 10 (103–109)	103 (98–111)	<0.001	109 ± 9 (106–112)	107 (103–117)	105 ± 9 (102–108)	105 (98–112)	<0.001
Endothelial function parameters										
ADMA (nmol/mL)	1.54 ± 1.39 (1.12–1.97)	0.94 (0.41–2.50)	1.47 ± 1.43 (1.04–1.91)	0.79 (0.41–2.35)	0.16	1.39 ± 1.43 (0.94–1.85)	0.56 (0.38–2.32)	1.52 ± 1.45 (1.06–1.98)	0.69 (0.38–2.67)	0.80
eNOS (U/L)	28.24 ± 30.27 (19.03–37.44)	18.74 (13.69–28.48)	27.03 ± 36.67 (15.88–38.18)	16.37 (13.55–25.16)	0.01	24.99 ± 24.63 (17.21–32.76)	17.56 (14.43–25.89)	25.86 ± 24.58 (18.10–33.62)	18.22 (13.79–28.61)	0.93
HCY (µmmol/L)	11.08 ± 2.79 (10.23–11.93)	11.18 (9.25–12.59)	10.72 ± 2.55 (9.95–11.50)	10.11 (8.99–12.56)	0.29	10.82 ± 3.03 (9.87–11.78)	10.25 (8.55–11.80)	10.86 ± 3.28 (9.82–11.89)	10.85 (8.99–12.20)	0.69
NO_2_ (µmol/L)	1.42 ± 0.30 (1.33–1.52)	1.34 (1.19–1.71)	1.47 ± 0.31 (1.38–1.57)	1.44 (1.26–1.73)	0.48	1.43 ± 0.30 (1.33–1.52)	1.39 (1.23–1.57)	1.48 ± 0.28 (1.39–1.56)	1.47 (1.30–1.62)	0.30
NO_3_ (µmol/L)	37.55 ± 18.52 (31.92–43.19)	31.37 (24.77–44.08)	41.71 ± 32.46 (31.84–51.58)	29.44 (25.57–43.23)	0.79	37.26 ± 16.22 (32.14–42.38)	32.46 (27.92–40.49)	42.57 ± 22.20 (35.57–49.58)	34.48 (27.38–56.65)	0.41
PAI-1 (ng/mL)	118.5 ± 32.4 (108.6–128.3)	119.6 (93.9–134.7)	118.9 ± 35.8 (108.0–129.7)	119.6 (91.2–137.8)	0.66	121.4 ± 42.0 (108.2–134.7)	117.0 (86.0–148.0)	118.5 ± 36.8 (106.9–130.1)	121.9 (89.2–145.1)	0.87
sVCAM-1 (ng/mL)	795.03 ± 190.49 (737.12–852.95)	738.00 (674.50–863.00)	821.86 ± 213.00 (757.10–886.62)	806.50 (684.50–914.50)	0.24	811.39 ± 268.55 (726.62–896.15)	777.00 (651.00–867.00)	843.33 ± 317.13 (743.24–943.44)	801.00 (719.00–911.00)	0.27
VEGF (pg/mL)	475 ± 268 (393–556)	408 (266–597)	459 ± 265 (379–540)	407 (257–566)	0.16	482 ± 256 (401–563)	432 (239–638)	492 ± 275 (405–579)	455 (250–660)	0.40
Arterial stiffness										
PWA SP (mmHg)	130 ± 12 (126–134)	130 (120–139)	125 ± 14 (120–129)	123 (117–132)	<0.001	130 ± 18 (125–136)	130 (121–137)	125 ± 11 (121–129)	126 (117–132)	0.06
PWA PP (mmHg)	42 ± 12 (38–45)	41 (36–47)	40 ± 10 (37–43)	39 (32–44)	0.10	42 ± 14 (38–47)	40 (35–48)	40 ± 9 (38–43)	40 (34–46)	0.80
PWA AIx	32 ± 14 (27–36)	30 (22–37)	29 ± 12 (25–32)	30 (21–35)	0.38	29 ± 14 (25–33)	29 (19–37)	34 ± 21 (27–41)	34 (24–41)	0.26
PWA AP (mmHg)	13 ± 6 (12–15)	13 (9–18)	12 ± 8 (10–15)	11 (8–15)	0.17	13 ± 7 (10–15)	12 (7–16)	15 ± 14 (11–20)	13 (7–20)	0.23
PWV (m/s)	7.1 ± 1.3 (6.7–7.6)	7.1 (6.2–7.9)	6.5 ± 0.8 (6.3–6.8)	6.5 (5.8–7.3)	<0.001	6.8 ± 1.2 (6.4–7.2)	6.6 (5.8–7.5)	6.7 ± 1.4 (6.2–7.2)	6.4 (5.7–7.4)	0.16
Antioxidant status parameters										
GSH (µmol/L)	22.71 ± 27.72 (14.29–31.14)	15.26 (10.34–24.52)	20.02 ± 28.54 (11.34–28.70)	12.90 (8.82–18.51)	0.003	28.42 ± 78.86 (3.53–53.31)	13.33 (7.02–24.36)	30.27 ± 85.48 (3.29–57.25)	16.27 (7.53–23.52)	0.22
SOD (U/mL)	3.81 ± 1.98 (3.20–4.41)	3.78 (2.60–4.59)	3.98 ± 2.12 (3.34–4.63)	3.78 (2.49–4.78)	0.50	3.26 ± 1.15 (2.90–3.62)	3.03 (2.35–4.19)	3.21 ± 1.63 (2.69–3.73)	2.76 (2.05–4.19)	0.78
TAS (ng/mL)	675 ± 872 (410–940)	252 (196–585)	601 ± 824 (348 to 854)	218 (180–381)	<0.001	672 ± 854 (402–941)	210 (179–605)	643 ± 879 (366–920)	194 (166–440)	0.63
Inflammatory markers										
hs-CRP (mg/L)	3.87 ± 3.76 (2.73–5.02)	2.75 (1.60–4.85)	4.34 ± 4.63 (2.94–5.75)	2.75 (1.60–4.85)	0.80	3.95 ± 3.85 (2.74–5.17)	2.30 (1.70–4.60)	4.22 ± 4.29 (2.86–5.57)	2.60 (1.70–4.20)	0.39
hs-IL-6 (pg/mL)	2.19 ± 1.54 (1.72–2.66)	1.85 (1.25–2.58)	2.34 ± 1.60 (1.86–2.83)	1.98 (1.24–3.14)	0.30	1.78 ± 0.91 (1.49–2.06)	1.50 (1.29–1.94)	2.16 ± 1.14 (1.80–2.53)	1.83 (1.51–2.64)	0.03
hs-IL-8 (pg/mL)	33.08 ± 26.3 (25.08–41.08))0	23.24 (17.35–42.94)	24.15 ± 24.73 (16.63–31.66)	17.52 (13.09–23.06)	0.009	32.64 ± 36.11 (21.25–44.04)	22.14 (12.72–29.08)	30.72 ± 29.51 (21.40–40.03)	18.47 (14.01–34.35)	0.82
MCP-1 (pg/mL)	427.72 ± 196.66 (367.93–487.51)	414.63 (300.73–550.72)	458.13 ± 219.54 (391.38–524.88)	453.85 (297.10–527.73)	0.07	425.55 ± 166.35 (373.04–478.05)	407.05 (324.65–495.35)	481.52 ± 119.58 (421.05–542.00)	465.90 (350.15–589.03)	0.007
MMP-2 (ng/mL)	209.03 ± 51.59 (193.34–224.71)	205.14 (169.00–238.09)	204.42 ± 53.10 (188.28–220.56)	205.04 (164.50–238.14)	0.36	201.97 ± 54.56 (184.75–219.20)	198.00 (169.00–219.00)	184.88 ± 47.97 (169.74–200.02)	175.96 (155.24–204.00)	0.003
MMP-9 (ng/mL)	759.7 ± 281.4 (674.2–845.3)	746.3 (567.0–936.0)	670.4 ± 263.3 (590.3–750.4)	628.3 (501.8–883.0)	0.04	778.0 ± 374.8 (659.7–896.3)	696.5 (521.0–918.0)	881.2 ± 450.3 (739.1–1023.4)	786.0 (577.3–962.0)	0.05
TNF-α (pg/mL)	40 ± 52 (24–56)	15 (12–33)	36 ± 48 (22–51)	14 (11–29)	0.003	38 ± 49 (22–53)	13 (11–33)	40 ± 51 (24–56)	12 (11–34)	0.63

ADMA—asymmetric dimethylarginine; BMI—body mass index; eNOS—endothelial nitric oxide synthase; GSH—glutathione; HCY—homocysteine; hs-CRP—high-sensitivity C reactive protein; hs-IL-6—high-sensitivity interleukin-6; hs-IL-8—high-sensitivity interleukin-8; MCP-1—monocyte chemo-tactic protein 1; MMP-2—matrix metalloproteinase-2; MMP-9—matrix metalloproteinase-9; NO2—nitrite; NO3—nitrate; PAI-1—plasminogen activator inhibitor-1; PWA AIx—augmentation index; PWA AP—augmentation pressure; PWA SP—central aortic systolic pressure; PWA PP—central aortic pulse pressure; PWV—pulse wave velocity; SD—standard deviation; SOD—superoxide dismutase; sVCAM-1—soluble vascular cell adhesion molecule-1; TAS—total antioxidant status; TNF-α—tumor necrosis factor alpha; VEGF—vascular endothelial growth factor; 95% CI—95% confidence interval.

**Table 3:** Comparison of the effects of endurance and endurance-strength training on endothelial function, arterial stiffness, oxidative stress, antioxidant status, and inflammatory markers.

	Endurance Training (*n* = 44)		Endurance-Strength Training (*n* = 41)		*p*
	Mean ± SD (95% CI)	Median (Q1–Q3)	Mean ± SD (95% CI)	Median (Q1–Q3)	
Anthropometric parameter					
Δ Weight (kg)	−1.2 ± 2.0 (−1.8–−0.5)	−1.0 (−2.3–−0.1)	−1.1 ± 2.8 (−2.0–−0.3)	−0.7 (−3.3–1.2)	0.80
Δ BMI (kg/m^2^)	−0.45 ± 0.78 (−0.68–−0.21)	−0.37 (−0.82–−0.05)	−0.46 ± 1.06 (−0.79–−0.12)	−0.31 (−1.33–0.45)	0.88
Δ Waist circumference (cm)	−4 ± 6 (−5–−2)	−4 (−7–−2)	−4 ± 5 (−5–−2)	−4 (−6–−2)	0.80
Endothelial function parameters					
Δ ADMA (nmol/mL)	−0.07 ± 0.29 (−0.15–0.02)	−0.03 (−0.18–0.06)	0.12 ± 0.79 (−0.13–0.37)	−0.02 (−0.06–0.11)	0.22
Δ eNOS (U/L)	−1.21 ± 9.37 (−4.05–1.64)	−0.70 (−3.47–0.58)	0.87 ± 9.0 (−1.98–3.72)	−0.75 (−2.87–2.89)	0.23
Δ HCY (µmmol/L)	−0.36 ± 1.88 (−0.93–0.21)	−0.28 (−1.39–0.97)	0.04 ± 1.98 (−0.59–0.66)	0.10 (−1.37–1.26)	0.29
Δ NO_2_ (µmol/L)	0.05 ± 0.41 (−0.08–0.17)	0.06 (−0.28–0.38)	0.05 ± 0.42 (−0.09–0.18)	0.02 (−0.11–0.33)	0.86
Δ NO_3_ (µmol/L)	4.15 ± 35.06 (−6.51–14.81)	0.10 (−6.58–5.99)	5.31 ± 23.87 (−2.22–12.85)	0.54 (−6.36–13.18)	0.76
Δ PAI-1 (ng/mL)	0.4 ± 26.1 (−7.5–8.3)	1.0 (−9.7–13.2)	−2.9 ± 27.4 (−11.60–5.72)	−3.0 (−7.0–12.0)	0.74
Δ sVCAM-1 (ng/mL)	26.83 ± 144.23 (−17.02–70.68)	21.50 (−60.75–114.75)	31.95 ± 146.05 (−14.15–78.05)	6.00 (−53.35–117.00)	0.98
Δ VEGF (pg/mL)	−15 ± 88 (−42–11)	−12 (−72–35)	10 ± 93 (−19–39)	9 (−22–56)	0.10
Arterial stiffness					
Δ PWA SP (mmHg)	−5 ± 11 (−8–−1)	−6 (−12–−1)	−5 ± 15 (−10–0)	−2 (−8–2)	0.20
Δ PWA PP (mmHg)	−2 ± 10 (−5–1)	−3 (−8–3)	−2 ± 14 (−6–2)	1 (−10–5)	0.43
Δ PWA AIx	−3 ± 15 (−8–1)	0 (−13–5)	5 ± 20 (−1–11)	2 (−5–12)	0.14
Δ PWA AP (mmHg)	−5 ± 9 (−8–2)	−4 (−9–0)	−9 ± 15 (−13–4)	−7 (−14–1)	0.16
Δ PWV (m/s)	−0.6 ± 1.2 (−1.0–0.2)	−0.4 (−0.8–0.0)	−0.2 ± 1.6 (−0.8–0.3)	−0.1 (−0.4–0.1)	0.06
Antioxidant status parameters					
Δ GSH (µmol/L)	−2.70 ± 6.08 (−4.54–−0.85)	−2.50 (−6.02–0.11)	1.85 ± 8.87 (−0.96–4.65)	0.74 (−1.99–4.65)	0.003
Δ SOD (U/mL)	0.18 ± 1.46 (−0.27–0.62)	−0.13 (−0.84–1.46)	−0.05 ± 1.31 (−0.47–0.36)	−0.07 (−0.84–0.96)	0.65
Δ TAS (ng/mL)	−74 ± 187 (−131–−17)	−29 (−65–−10)	−29 ± 265 (−112–55)	−2 (−28–18)	0.002
Inflammatory markers					
Δ hs-CRP (mg/L)	0.47 ± 2.86 (−0.40–1.34)	−0.08 (−1.45–1.25)	0.27 ± 2.99 (−0.68–1.21)	−0.30 (−1.00–0.60)	0.60
Δ hs-IL-6 (pg/mL)	0.16 ± 1.79 (−0.39–0.70)	0.30 (−0.41–0.78)	0.39 ± 1.11 (0.04–0.74)	0.36 (−0.23–0.70)	0.51
Δ hs-IL-8 (pg/mL)	−8.93 ± 36.93 (−20.16–2.30)	−3.75 (−18.37–1.83)	−1.93 ± 47.51 (−16.92–13.07)	−1.07 (−8.07–8.93)	0.11
Δ MCP-1 (pg/mL)	30.41 ± 98.70 (0.40–60.42)	27.15 (−38.28–83.45)	55.98 ± 123.00 (−17.15–94.80)	48.75 (−36.25–126.75)	0.38
Δ MMP-2 (ng/mL)	−4.61 ± 27.68 (−13.02–3.81)	−2.97 (−23.02–15.61)	−17.10 ± 36.06 (−28.48–−5.71)	−15.00 (−34.00–3.00)	0.07
Δ MMP-9 (ng/mL)	−89.4 ± 258.4 (−167.9–−10.8)	−75.0 (−267.2–103.8)	103.2 ± 329.3 (−0.7–207.2)	117.0 (−63.3–254.0)	0.003
Δ TNF-α (pg/mL)	−3 ± 12 (−7–0)	−1 (−3–0)	2 ± 16 (−3–7)	0 (−1–1)	0.07

ADMA—asymmetric dimethylarginine; BMI—body mass index; eNOS—endothelial nitric oxide synthase; GSH—glutathione; HCY—homocysteine; hs-CRP—high-sensitivity C reactive protein; hs-IL-6—high-sensitivity interleukin-6; hs-IL-8—high-sensitivity interleukin-8; MCP-1—monocyte chemo-tactic protein 1; MMP-2—matrix metalloproteinase-2; MMP-9—matrix metalloproteinase-9; NO2—nitrite; NO3—nitrate; PAI-1—plasminogen activator inhibitor-1; PWA AIx—augmentation index; PWA AP—augmentation pressure; PWA SP—central aortic systolic pressure; PWA PP—central aortic pulse pressure; PWV—pulse wave velocity; SD—standard deviation; SOD—superoxide dismutase; sVCAM-1—soluble vascular cell adhesion molecule-1; TAS—total antioxidant status; TNF-α—tumor necrosis factor alpha; VEGF—vascular endothelial growth factor; 95% CI—95% confidence interval.

## Data Availability

The data presented in this study are available on request from the corresponding author. The data are not publicly available due to the disagreement of the study participants.

## Supplementary Materials

The following are available online at https://www.mdpi.com/article/10.3390/jcm10081639/s1, Table S1: CONSORT 2010 checklist of information to include when reporting a randomized trial.
